# Antiobesity Effects of the Ethanol Extract of *Laminaria japonica* Areshoung in High-Fat-Diet-Induced Obese Rat

**DOI:** 10.1155/2013/492807

**Published:** 2013-01-10

**Authors:** Woong Sun Jang, Se Young Choung

**Affiliations:** Department of Preventive Pharmacy and Toxicology, College of Pharmacy, Kyung Hee University, Seoul 130-701, Republic of Korea

## Abstract

*Laminaria japonica* Areshoung, a widely consumed marine vegetable, has traditionally been used in Korean maternal health. The present study investigated the antiobesity effects of *Laminaria japonica* Areshoung ethanol extract (LE) and its molecular mechanism in high-fat-diet-induced obese rats. Six-week-old Sprague-Dawley male rats were separately fed a normal diet or a high-calorie high-fat diet for 6 weeks; then they were treated with LE or tea catechin for another 6 weeks. LE administration significantly decreased the body weight gain, fat-pad weights, and serum and hepatic lipid levels in HD-induced obese rats. The histological analysis revealed that LE-treated group showed a significantly decreased number of lipid droplets and size of adipocytes compared to the HD group. To elucidate the mechanism of action of LE, the levels of genes and proteins involved in obesity were measured in the liver and skeletal muscle. LE treatment resulted in an increased expression of fatty acid oxidation and thermogenesis-related genes in obese rats. Conversely, the expression of the fat intake-related gene (ACC2) and lipogenesis-related genes was reduced by LE treatment. Additionally, LE treatment increased the phosphorylation of AMP-activated protein kinase and its direct downstream protein, acetyl coenzyme A carboxylase, which is one of the rate-limiting enzymes in fatty acid synthesis pathway. These findings demonstrate that LE treatment has a protective effect against a high-fat-diet-induced obesity in rats through regulation of expression of genes and proteins involved in lipolysis and lipogenesis.

## 1. Introduction 

Obesity is a chronic metabolic disorder that results from the imbalance between energy intake and energy expenditure. It is characterized by enlarged fat mass and elevated lipid concentration in blood [1, 2]. On a global scale, obesity has reached epidemic proportions and is a major contributor to the global burden of chronic disease and disability. Currently, more than one billion adults worldwide are overweight and at least 300 million of them are clinically obese [3]. Importantly, obesity is often associated with a variety of chronic diseases such as hyperlipidemia, diabetes mellitus, hypertension coronary artery disease, and certain cancers [4–7]. Therefore, prevention and treatment of obesity are important for a healthy life [8].

Although a number of pharmacological approaches to the treatment of obesity have been recently investigated, only a few drugs have been approved for clinical usage. Current therapies for obesity treatment include the reduction of nutrient absorption and the administration of drugs that affect lipid mobilization and utilization (e.g., orlistat and sibutramine) [9]. However, owing to the adverse side effects associated with many antiobesity drugs, more recent trials have focused on screening natural sources that have been reported to reduce body weight with minimal side effects [10]. This may be an excellent alternative strategy for developing effective and safe antiobesity drugs in the future [11–13]. 

A variety of natural products, including crude extracts and isolated compounds from plants, have been widely used traditionally to treat obesity [14–16]. A wealth of information indicates that numerous bioactive components from nature are potentially useful in obesity treatments. A good example of this is polyphenolic compounds showing strong antiobesity activity including apigenin, genistein, and the catechins [16–18]. 

*Laminaria japonica* Areshoung is widely consumed as a marine vegetable and has been used to promote maternal health in Korea. Recently, it has been reported that *Laminaria japonica* Areshoung possesses various biological functions including anti-inflammatory [19], antitumor [20], antiatheroscloresis [21], and antidiabetic activity [22]. It has been reported that ethanol extracts from *Laminaria japonica* Areshoung contain mannitol, iodine, micro elements, free amino acids, glycolipids, polyunsaturated fatty acids, fucosterine, polyphenols, and fucoxanthin [23]. In addition, these compounds from ethanol extracts of *Laminaria japonica* Areshoung have shown antiallergenic activities [24]. However, little is known about the effects of *Laminaria japonica* Areshoung on obesity in animal model. 

In the present study, we investigated the antiobesity effects of ethanol extract of *Laminaria japonica* Areshoung (LE) in high-fat diet-(HD-) induced obese rats. Body weight gain, food intake, fat-pad weights, and serum and hepatic lipid levels were measured. Green tea catechin, which has shown antiobesity [25, 26], anti-diabetic [27], and cardioprotective effects [28, 29] in animal and human studies, was used as a positive control. To gain insight into the molecular mechanisms underlying the effects described above, we investigated the expression of genes and proteins related to lipid metabolism in LE-treated obese rats. Our results demonstrate the great potential of LE for the treatment of obesity.

## 2. Materials and Methods

### 2.1. Experimental Material Preparation

#### 2.1.1. Preparation of *L. japonica*

*L. japonica* was collected from Gijang aquaculture farm, Busan, Republic of Korea, in May 2010, and a voucher specimen was deposited in the laboratory (H. R. Kim). Samples were rinsed with tap water to remove salt and dried in an air dryer at 60°C for 40 h. A dried sample was ground with a hammer mill, and the powder was stored at −20°C until used.

#### 2.1.2. Extraction of *L. japonica* and Liquid Chromatography

**Figure 1:**
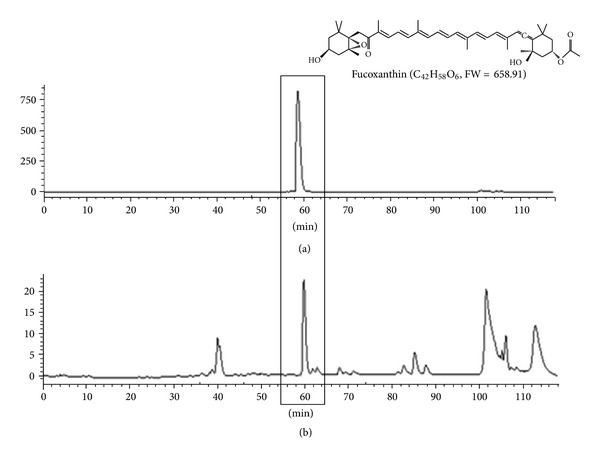
HPLC chromatograms at 410 nm of fucoxanthin standard (a) and ethanol extract of *L*. *japonica* (b). The main peak (retention time = 60 min) of the ethanol extract of *L*. *japonica* at 410 nm was observed at the same position as that of the fucoxanthin standard. Main peak of the extract was identified as fucoxanthin.

Dried powder (2.5 kg) of *Laminaria japonica* Areshoung was extracted three times with 96% (v/v) ethanol (EtOH) for 3 h at 70°C. The combined extracts were filtered and concentrated under reduced pressure to obtain the EtOH extracts (446.6 g). Ethanol extract of *Laminaria japonica* Areshoung was analyzed by Shimadzu high-performance liquid chromatography (HPLC) system with Luna RP-18 Luna C18(2), 5 *μ*m, 250 × 10 mm, Phenomenex, Torrance, CA, USA. The analysis of LE was conducted using acetonitrile and 0.1% formic acid in water as the mobile phase. The flow rate was 1 mL/min at 35°C oven temperature, and detection was performed at 410 nm (Figure 1). The main peak (retention time = 60 min) of ethanol extract of *L. japonica* at 410 nm was observed at the same position as that of the fucoxanthin standard (Figure 1(a)). These results suggest that ethanol extract of *L. japonica* mainly includes fucoxanthin.

### 2.2. Animals and Experimental Diets

Sixty 6-week-old male Sprague-Dawley rats (Koatech, Pyeongtaek, Republic of Korea) were individually housed in standard cages and placed in a room where the temperature was kept at 21 ± 2.0°C, the relative humidity at 50 ± 5%, and the light on a 12 h light/dark cycle. All the rats consumed a commercial diet for 1 week. After that, the animals were separately fed a normal diet (ND) or high-fat diet (HD) (Research Diets Inc., New Brunswick, USA) for 6 weeks. Then the animals were subdivided into six groups (*n* = 6)—ND group, HD group, HD + LE (100, 200, or 400 mg/kg) groups, and HD + tea (tea catechin, 100 mg/kg) group—and treated with LE or tea catechin for another 6 weeks. Tea catechin was kindly provided by Amore Pacific Corp. (Seoul, Republic of Korea). Diet compositions are shown in Table 1. Food intake was recorded daily, and body weights were monitored every 2 days during the feeding period. At the end of the treatment period, rats were subjected to fasting for 12 hours. After anesthetization with diethyl ether, the epididymal, abdominal, visceral, and brown fat-pads and livers were removed from rats, rinsed with phosphate-buffered saline, and then weighed. The liver and fat pad samples were stored at −70°C until analysis. This study was approved by the Institutional Animal Care and Use Committee of Kyung Hee University, Seoul.

### 2.3. Biochemical Analysis

At the end of treatment, biochemical analysis was performed after 12 h fasting. Blood was drawn from the inferior vena cava into a heparin-coated tube, and the plasma was obtained by centrifuging the blood at 15.000 g for 15 min at 4°C. The plasma triglyceride concentration was measured using a kit based on a lipase-glycerol phosphate oxidase method (Asan Pharmaceutical, Seoul, Republic of Korea). Plasma total cholesterol (TC), triglyceride (TG), high-density lipoprotein cholesterol (HDL-C) and free fatty acid (FFA) concentrations were determined using a commercial kit (Asan Pharmaceutical, Seoul, Republic of Korea). Low-density lipoprotein-cholesterol (LDL-C) was calculated using the following equation; LDL-C = TC − HDL-C − TG/2.18 [30]. Free fatty acid (nonesterified fatty acid, NEFA) was also measured using NEFA-Wako (Wako Pure Chemical Industries, Osaka, Japan). Plasma glutamic oxaloacetic transaminase (GOT), glutamic pyruvic transaminase (GPT) and blood urea nitrogen (BUN) levels were measured using a commercial kit (Asan Pharmaceutical, Seoul, Republic of Korea). Plasma adiponectin, leptin, glucose, tumor necrosis factor-*α* (TNF-*α*), and insulin levels were analyzed using enzyme-linked immunosorbent assay kits (Shibayagi, Gunma, Japan).

### 2.4. Hepatic Lipid Profiles

Hepatic lipids were extracted using the method developed by Folch et al. [31], and the dried lipid residues were dissolved in 1 mL ethanol. High-density lipoprotein cholesterol (HDL-C), TC, TG, and FFA concentrations in the hepatic lipid extracts were measured using the same enzymatic kits that were applied for the plasma analysis.

### 2.5. Oil-Red O Staining

The tissues were sliced (7 *μ*m) and stained in 60% of the oil red O stock solution (0.5 g oil red O in 100 mL isopropanol) for 30 min and then briefly washed with 60% isopropanol and then with distilled water for microscopic observation and photography.

### 2.6. Hematoxylin and Eosin Staining

Tissue samples of liver and epididymal fat pads were fixed with 4% buffered formalin and embedded in paraffin. Standard 4 *μ*m thick sections were stained with hematoxylin and eosin, viewed with an optical microscope (Olympus Optical, Tokyo, Japan), and photographed at a final magnification of 200x. Total 48 planes (8 planes per group) were used to determine the size of adipocytes. The average size of adipocytes was measured by using Image J software (National Institute of Mental Health, Bethesda, USA).

### 2.7. RNA Isolation from Tissue Samples

Livers, skeletal muscles, and adipose tissues were dissected, weighed, frozen in liquid nitrogen, and stored at −70°C until use. The tissue samples (in the amount of 100 mg) were homogenized in easy blue reagent (iNtRON, Gyeonggi, Korea). Phase separation of RNA was performed by adding one-fifth volume of chloroform and centrifugation at 12,000 g for 10 min. Isopropyl alcohol (0.5 mL) was added to the aqueous phase to precipitate total RNA, then RNA pellet was washed twice with 75% ethanol. The RNA sample was dried and dissolved in TE buffer (10 mM Tris, bring to pH 8.0 with HCl, 1 mM EDTA). RNA concentration was determined by measuring absorbance at 260 and 280 nm using an Optizen 2120UV spectrophotometer (Mecasys, Daejeon, Republic of Korea).

### 2.8. Reverse Transcription-Polymerase Chain Reaction Analysis

Reverse transcription- (RT-) polymerase chain reaction (PCR) assays for mRNA levels in the skeletal muscle, liver, and adipose tissue were performed using a Maxime RT-PCR PreMix kit (iNtRON, Gyeonggi, Republic of Korea) according to the manufacturer's protocol. RT was performed at 45°C for 30 minutes. PCR was carried out as follows: 5 minutes at 94°C, 30 cycles of 94°C for 1 minute, 56°C for 1 minute, and 72°C for 5 minutes, and a 5-minute incubation at 72°C. The *β*-actin gene was used as an internal control. The sequences of primes used in this study are shown in Table 2.

### 2.9. Protein Extraction and Western Blot Analysis

Liver tissues (100–150 *μ*g) were homogenized in extraction buffer (100 mM Tris-HCl, pH 7.4, 5 mM EDTA, 50 mM NaCl, 50 mM sodium pyrophosphate, 50 mM NaF, 100 mM orthovanadate, 1% Triton X-100, 1 mM PMSF, 2 *μ*g/mL aprotinin, 1 *μ*g/mL pepstatin A, and 1 *μ*g/mL leupeptin). Tissue homogenates were centrifuged at 13,000 g for 20 min at 4°C, and the resulting supernatants were used for western blot analysis. The protein concentrations of supernatants were measured by the Lowry assay (Bio-Rad, Hercules, CA, USA). Protein samples (50 *μ*g/lane) were separated by 8% SDS-PAGE and were transferred onto the nitrocellulose membrane (Amersham Biosciences, Buckinghamshire, UK). After blocking (5% nonfat dry milk in 10 mmol/L Tris, pH 7.5, 100 mmol/L NaCl, and 0.1% Tween 20) for 2 hours at room temperature, the membrane was incubated overnight at 4°C with primary antibodies (1 : 1,000 dilution). Antibodies against acetyl-CoA carboxylase (ACC), phospho-ACC at Ser 79, AMP-activated protein kinase *α* (AMPK*α*), and phospho-AMPK *α* at Thr 172 were purchased from Cell Signaling Technology (Beverly, USA). After the membrane was incubated with 1 : 5,000-diluted secondary antibody for 1 hour, immune reactive signals were detected by chemiluminescence (ECL) detection system (Fuji Film, Tokyo, Japan). The densities of bands were measured by science lab 2.0 software (Fuji Film, Tokyo, Japan).

### 2.10. Statistical Analysis

Numerical data are expressed as means ± standard deviation values. The significance of differences was examined using ANOVA, followed by Tukey's test. Differences between groups were considered statistically significant at *P* < 0.05. 

## 3. Results

### 3.1. Effect of LE Treatment on Body Weight, Food Efficiency Ratio, and Fat-Pad Weights

**Figure 2:**
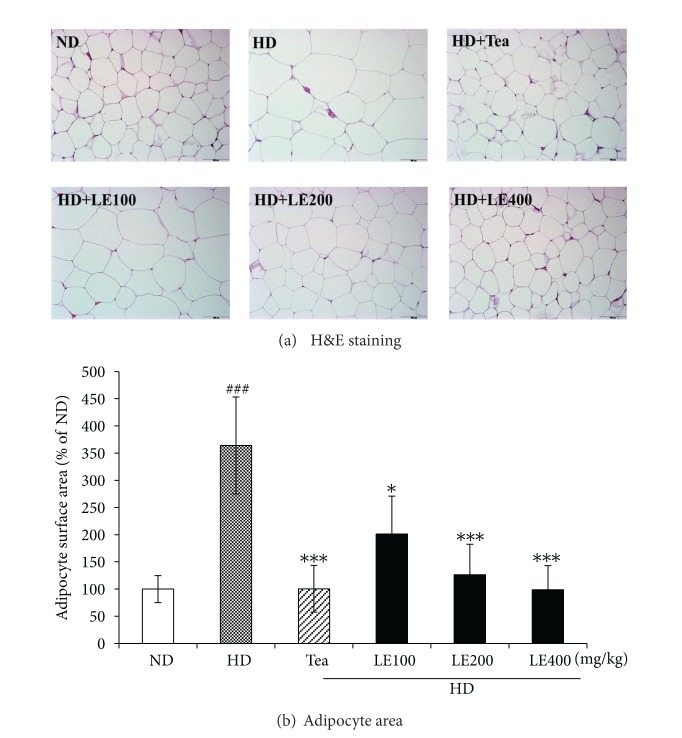
Histological analysis of epididymal adipose tissue (a) and adipocyte surface area (b) of the rats fed the experimental diets for 6 weeks. All sections were stained with hematoxylin and eosin; magnification, ×200. Magnification bar = 100 *μ*m. Mean surface area for epididymal white adipocytes was measured using Image J software. Data are mean ± SD (*n* ≥ 8). ^###^ *P* < .001 versus the ND group; *P < .05, ***P < .001 versus HD group.

The body weight and food intakes are shown in Table 3. During the 6 weeks experimental period, the amount of food efficiency ratio as well as body weight changes was measured every 2 days. The final body weight and weight gain of the HD group were 30.1% and 65.9% greater, respectively, than those of the ND group. LE administration suppressed HD-induced body weight increase in a dose-dependent manner. The LE (400 mg/kg) and tea catechin supplementation significantly reduced body weight gain compared to no treatment (HD group) by 47%, respectively. The food intake and food efficiency ratio (FER) of the obese rats given LE were significantly lower than the values for the rats fed HD only, suggesting that LE regulates food intake and energy metabolism. In addition, the epididymal, abdominal, visceral, and brown fat-pad weights were significantly higher in the HD group than the value for the ND group, and these increases were lower by LE treatment (Table 3). In agreement with these results, the size of adipocytes from the epididymal white adipose tissue of the obese rats fed LE (400 mg/kg) was 72.9% smaller than those for the HD group (Figure 2).

### 3.2. Effect of LE Treatment on Lipid Parameters in Blood

**Figure 3:**
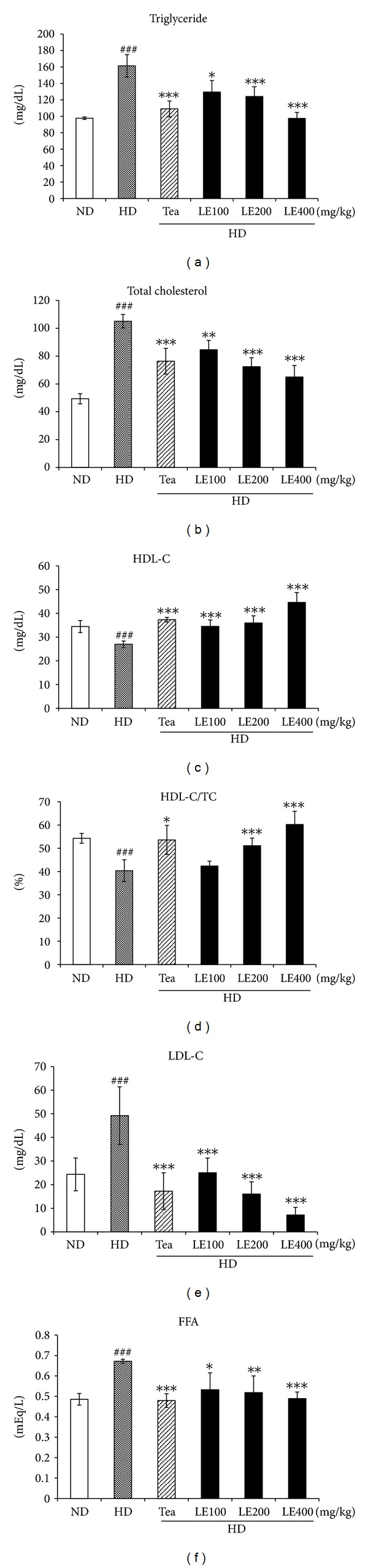
Effect of LE on lipid levels in blood. FFA, free fatty acid; HDL-C, high-density lipoprotein-cholesterol; LDL-C, low-density lipoprotein-cholesterol. Values are mean ± SD (*n* ≥ 8). ^##^ *P* < .01, ^###^ *P* < .001 versus the ND group; *P < .05, **P < .01, ***P < .001 versus HD group.

Rats in the HD group exhibited significantly higher TG, TC, LDL-C and FFA levels and lower HDL-C, and HDL-C/TC ratio, compared to rats in the ND group (Figure 3). However, LE (400 mg/kg) or tea catechin treatment led to a reversal of the aforementioned parameters to the levels similar to those of the ND group. LE treatment (400 mg/kg) resulted in 39.6%, 38.1%, 85.5%, and 27.2% decreases in serum TG, TC, LDL-C, and FFA, respectively, and a 65.7% and 49.3% increase in HDL-C and HDL-C/TC ratio, respectively, compared with no treatment (HD group). These results indicate that oral administration of LE suppresses the accumulation of body fat, resulting in improved lipid profiles in serum. 

**Figure 4:**
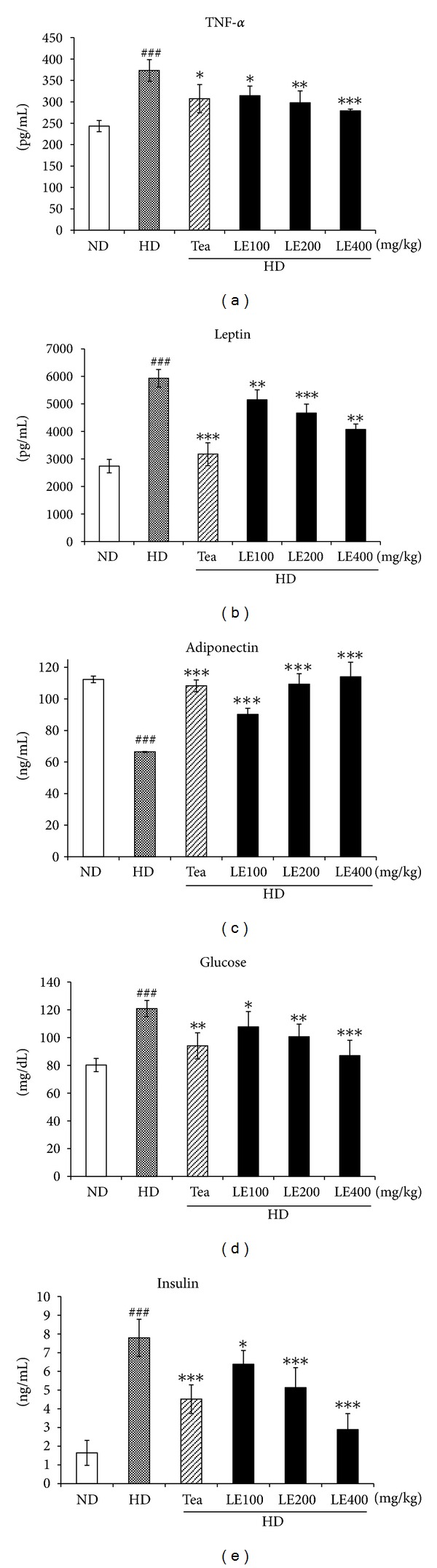
Effect of LE on the level of tumor necrosis factor-*α*, leptin, adiponectin, glucose, and insulin in blood. Values are mean ± SD (*n* ≥ 8). ^###^ *P* < .001 versus the ND group; *P < .05, **P < .01, ***P < .001 versus HD groups. ^###^ *P* < .001 versus the ND group; *P < .05, **P < .01, ***P < .001 versus HD groups.

Serum glucose, insulin, leptin, and TNF-*α* levels were also increased by high-fat-diet (Figure 4). Conversely, the adiponectin level was significantly lower in the HD group. However, LE or tea catechin supplemented showed decreased TNF-*α*, leptin, glucose, and insulin levels, and increased adiponectin level in blood compared to the HD group. TNF-*α* mRNA level in adipose tissue was also decreased by LE treatment (see Supplementary in Figure  1). To evaluate the effect of LE on hepatic and renal function, we determined the plasma GOT, GPT and BUN level in LE-treated obese rat (Table 4). Although serum levels of GOT, GPT, and BUN of the HD group were significantly higher than those of the ND group, and tea catechin or LE treatment reduced the serum GOT and BUN levels to values of the ND groups, they all were within normal range. These results indicate that LE supplementation has no liver and kidney toxicity. 

### 3.3. Effect of LE Treatment on Hepatic Lipid Profiles

**Figure 5:**
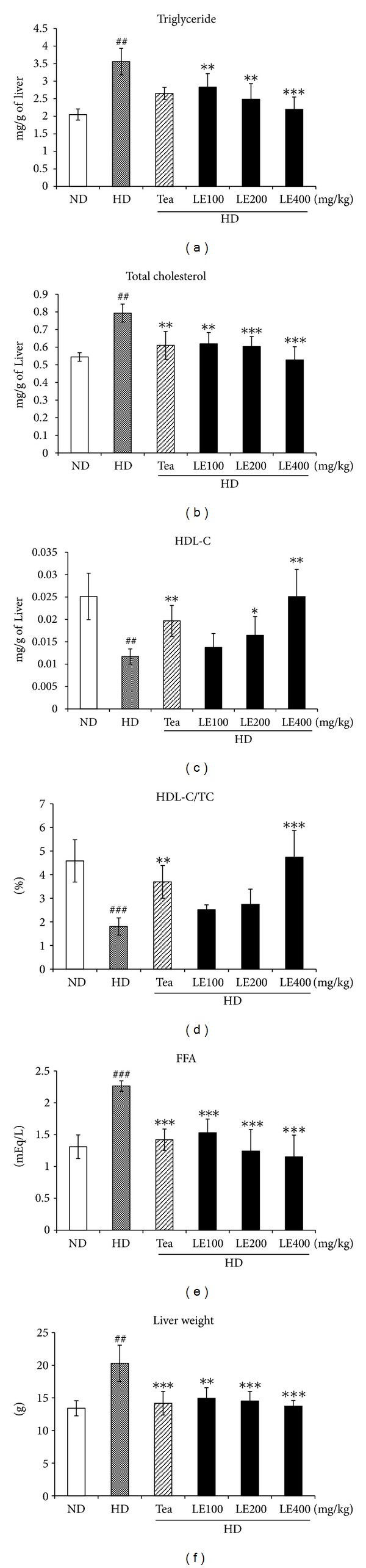
Effect of LE on liver weight and lipid levels in liver. Values are mean ± SD (*n* ≥ 8). ^##^ *P* < .01, ^###^ *P* < .001 versus the ND group; *P < .05, **P < .01, ***P < .001 versus HD groups.

**Figure 6:**
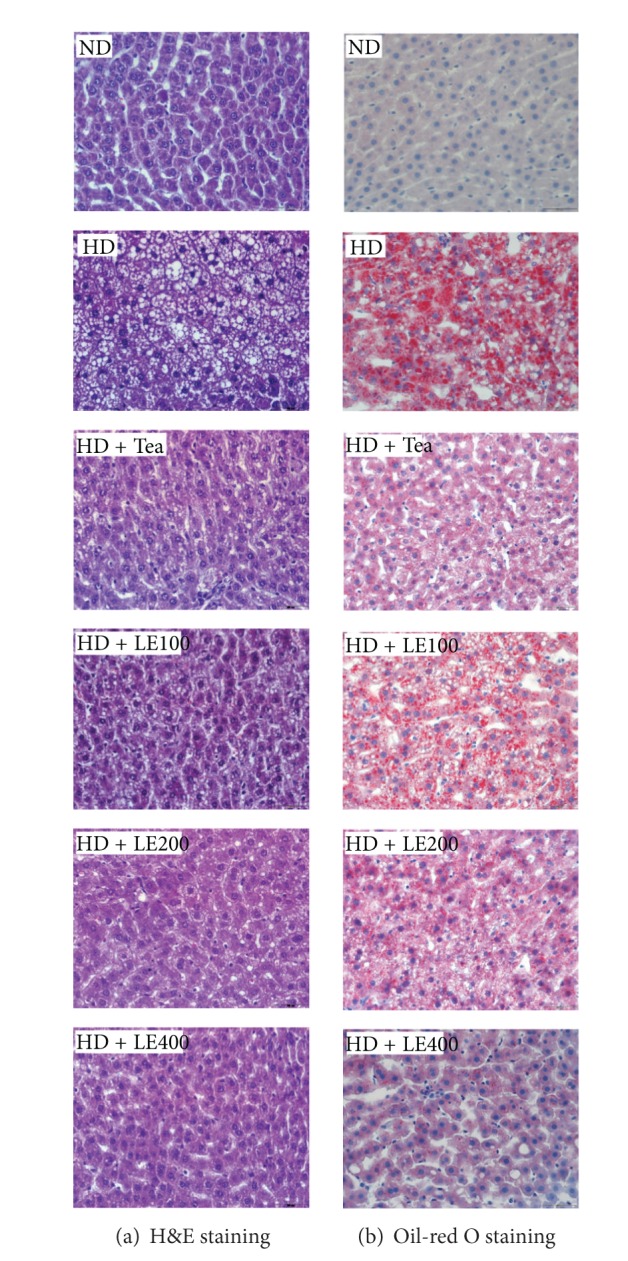
Effect of LE on lipid accumulation in liver. (a) H&E staining and (b) oil-red O staining (×200). Eight sections per group were measured. Rats were given an ND, an HD, HD containing LE or HD containing tea catechin for 6 weeks. The liver was removed, fixed in 10% formalin, paraffinized, and stained using hematoxylin and eosin (a). A cryostat was also used to prepare tissue sections for staining with oil-red O (b). Details of the diets are given in Section 2.

Liver weights and hepatic lipid levels are shown in Figure 5. Liver weight was about 1.5 times greater in the HD group than that in the ND group. In addition, hepatic TG, TC, and FFA levels in the HD group were significantly higher than ND group. Conversely, HDL-C and HDL-C/TC ratio levels in liver were significantly lower than those in the ND group. LE-treated group (400 mg/kg) resulted in 38.3%, 33.5%, and 49.1% decreases in hepatic TG, TC, and FFA, respectively, and 114.4% and 163.1% increase in HDL-C and HDL-C/TC ratio, respectively, compared with the HD group. Consistent with these findings, H&E sections from the rats fed HD only revealed the presence of a large number of circular lipid droplets which were also detected by oil-red O staining (Figure 6). These lipid droplets were strikingly reduced both in size and number in the liver of LE or tea catechin-treated rats, suggesting that LE treatment effectively inhibits lipid accumulation in liver.

### 3.4. Effect of LE Treatment on the Expression of Genes Related to Lipid Metabolism in Skeletal Muscle

**Figure 7:**
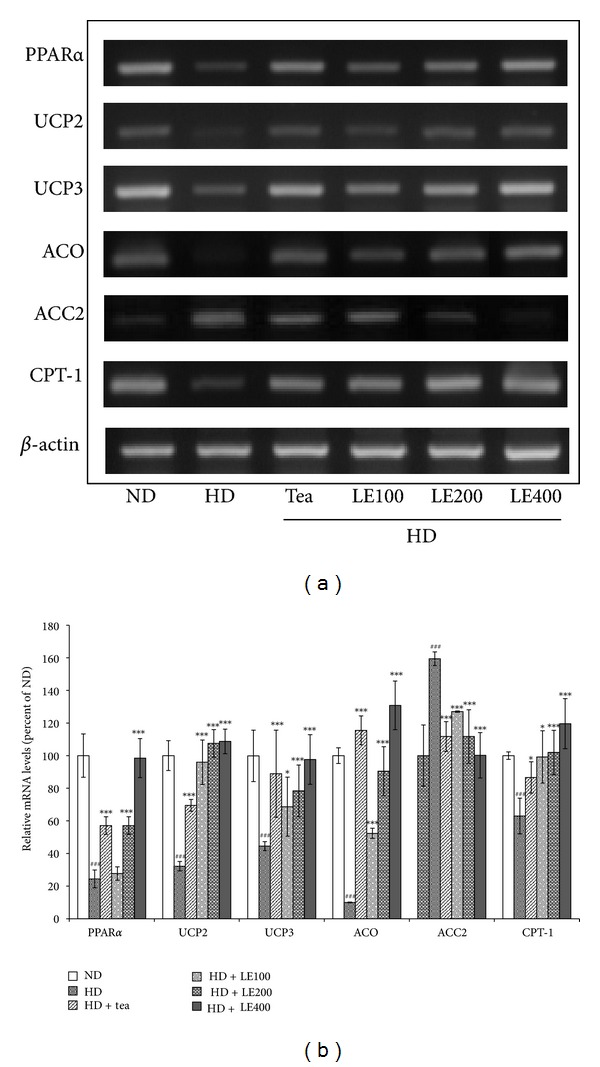
Effect of LE on the gene expressions related to lipid metabolism in skeletal muscle. Rats were treated with LE or tea catechin for 6 weeks. The level of PPAR*α*, UCP2, UCP3, ACO, ACC2, and CPT-1 mRNA was determined by RT-PCR analysis. Actin mRNA was used as an internal control. Relative fold intensities were analyzed using band densities obtained from RT-PCR. All RNA levels were normalized to that of actin mRNA. Results shown are mean and standard deviation (*n* ≥ 8). ^###^ *P* < .001 versus the ND group; *P < .05, ***P < .001 versus HD groups.

RT-PCR was performed to measure the mRNA expression of fatty acid oxidation-related genes such as acetyl-CoA carboxylase 2 (ACC2), carnitine palmitoyltransferase 1 (CPT-1), and lipolytic genes such as peroxisome proliferator-activated receptor *α* (PPAR*α*), acyl CoA oxidase (ACO), uncoupling protein 2 (UCP2), and uncoupling protein 3 (UCP3) in skeletal muscle which is an important organ for energy expenditure (Figure 7). Compared with control rats (HD group), LE-treated rats showed significantly induced mRNA expression of PPAR*α*, ACO, CPT1, UCP2 and UCP3, which were highly reduced in HD rats. Tea catechin as a positive control also showed similar effects of LE treatment (200 mg/kg). These results indicate that oral administration of LE enhanced the expression of fatty acid oxidation-related genes and lipolytic genes in a dose-dependent manner.

### 3.5. Effect of LE Treatment on the Expression of Genes Related to Lipid Metabolism in Liver

**Figure 8:**
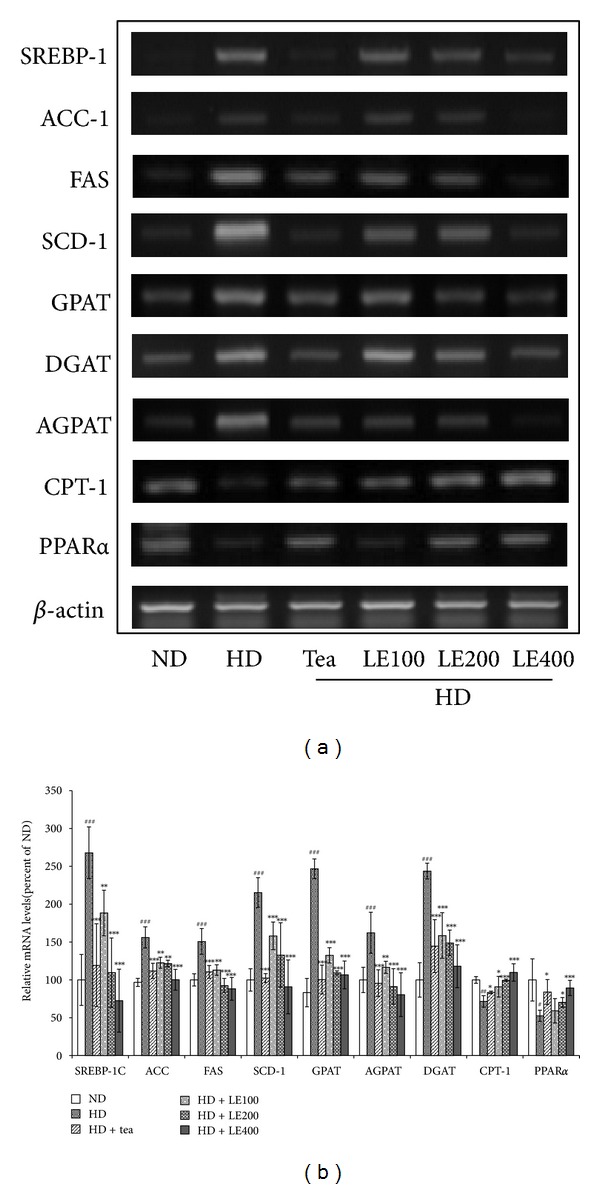
Effect of LE on the gene expressions related to lipid metabolism in liver. Rats were treated with LE or tea catechin for 6 weeks. The level of SREBP-1, ACC, FAS, SCD-1, GPAT, DPAT, AGPAT, CPT-1 and PPAR*α* mRNA were determined by RT-PCR analysis. Actin mRNA was used as an internal control. Relative fold intensities were analyzed using band densities obtained from RT-PCR. All RNA levels were normalized to that of Actin mRNA. Results shown are mean and standard deviation (*n* ≥ 8). ^#^ *P* < .05, ^##^ *P* < .01, ^###^ *P* < .001 versus the ND group; *P < .05, **P < .01, ***P < .001 versus HD groups.

Expressions of lipogenesis-related genes (SREBP-1c, ACC, FAS, SCD-1, and GPAT, AGPAT, DGAT) and fatty acid oxidation-related genes (CPT-1 and PPAR*α*) in liver were measured by RT-PCR (Figure 8). mRNA expressions of SREBP-1c, ACC, FAS, SCD-1, GPAT, AGPAT, and DGAT in the HD group were significantly higher than those in the ND group. However, tea catechin or LE (400 mg/kg) supplementation significantly suppressed the mRNA expressions to near-normal levels. Conversely, mRNA expressions of PPAR*α* and CPT1 in high-fat diet rats were significantly rescued by LE treatment. Tea catechin also showed similar effects to LE (400 mg/kg).

### 3.6. Effect of LE Treatment on the Expression of Genes Related to Lipid Metabolism in Brown and White Adipose Tissue

**Figure 9:**
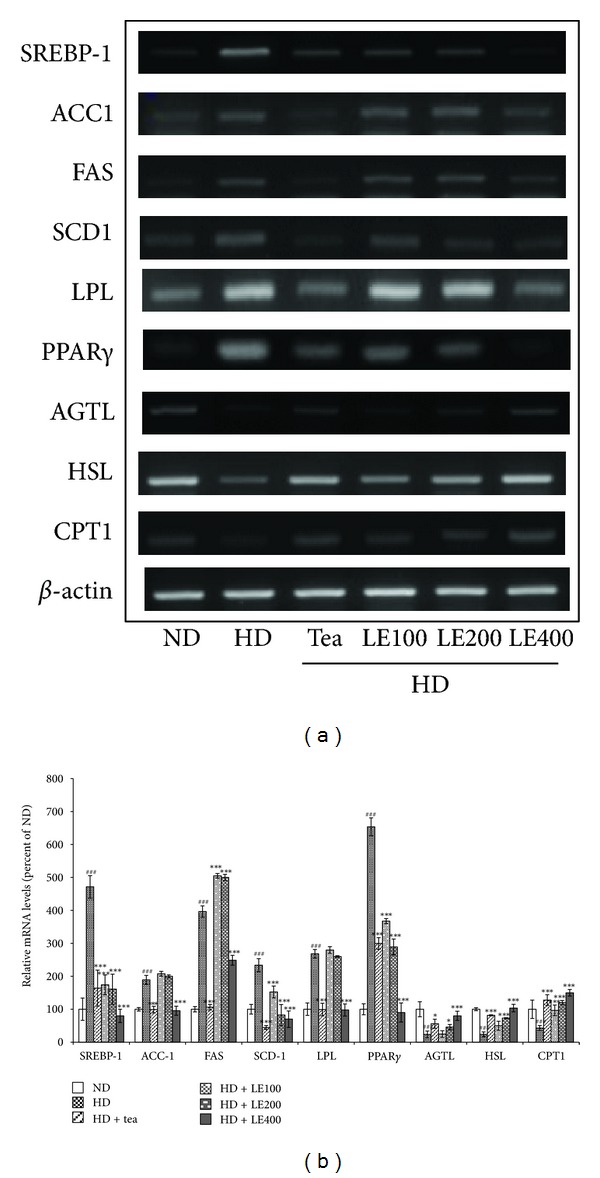
Effect of LE on the gene expressions related to lipid metabolism in brown and white adipose tissues. Rats were treated with LE or tea catechin for 6 weeks. The level of SREBP-1, ACC1, FAS, SCD-1, LPL, PPAR*γ*, ATGL, HSL, CPT-1, and UCP1 mRNA was determined by RT-PCR analysis. Actin mRNA was used as an internal control. Relative fold intensities were analyzed using band densities obtained from RT-PCR. All RNA levels were normalized to that of actin mRNA. Results shown are mean and standard deviation (*n* ≥ 8). ^#^ *P* < .05, ^##^ *P* < .01, ^###^ *P* < .001 versus the ND group; *P < .05, **P < .01, ***P < .001 versus HD groups.

Expressions of lipogenesis-related genes (SREBP-1c, ACC, FAS, and SCD-1), fatty acid uptake-related genes (LPL, and PPAR*γ*), lipolysis-related genes (ATGL and HSL), and a fatty acid oxidation-related gene (CPT-1) in epididymal white adipose tissue and a thermogenesis-related gene (UCP1) in brown adipose tissue were measured by RT-PCR (Figure 9). mRNA expressions of SREBP-1c, ACC, FAS, SCD-1, LPL, and PPAR*γ* in the HD group were significantly higher than those in the ND group. However, tea catechin or LE (400 mg/kg) supplementation significantly suppressed the mRNA expressions to near-normal levels. Conversely, mRNA expressions of ATGL, HSL, UCP1, and CPT1 in high fat diet rats were significantly rescued by LE treatment. Tea catechin also showed similar effects to LE (400 mg/kg).

### 3.7. Effect of LE Treatment on the Activity of AMPK and ACC in Liver

**Figure 10:**
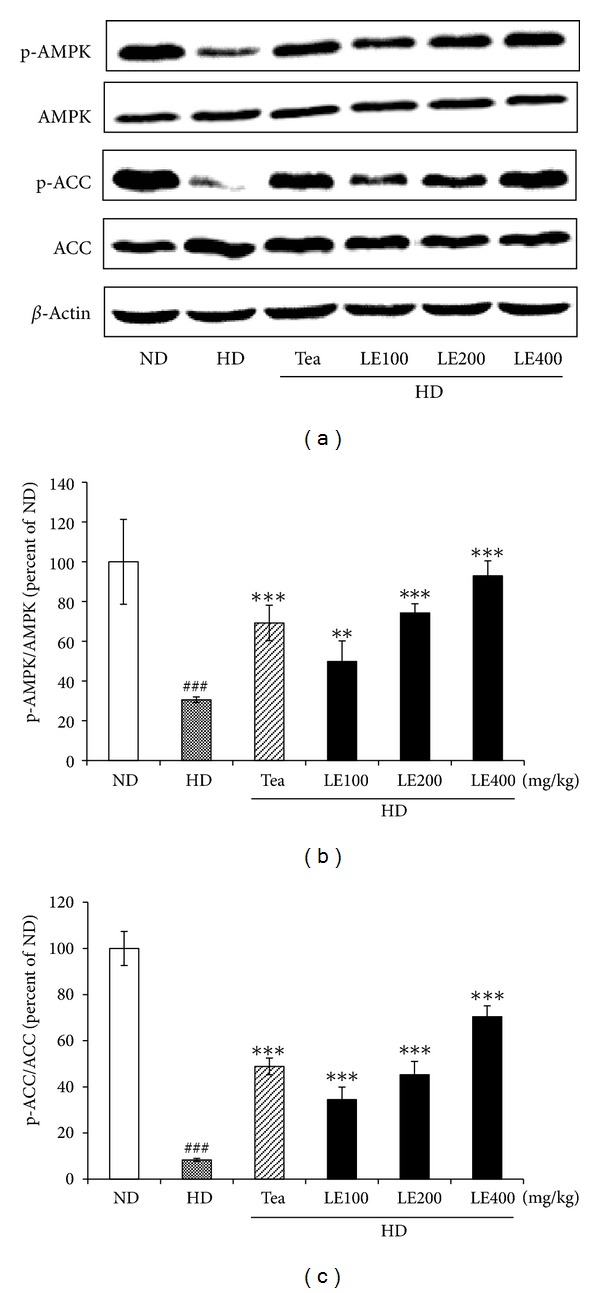
Effect of LE on the phosphorylation of AMPK and ACC in liver. LE treatment enhanced phosphorylation in a dose-dependent manner. Rats were treated with LE or tea catechin for 6 weeks. Relative fold intensities were analyzed using band densities obtained from western immunoblotting. All levels were normalized to that of *β*-actin. Total proteins were extracted by cell lysis buffer and resolved by SDS-PAGE. Results shown are mean and standard deviation (*n* ≥ 6). ^###^ *P* < .001 versus the ND group; **P < .01, ***P < .001 versus HD groups.

To determine whether LE supplementation affects the activity of AMPK, which is one of the major regulators in lipid metabolism, western immunoblotting analysis was performed using the whole tissue extract prepared from the liver of LE-treated rats. As shown in Figure 10, feeding the rats with the HD significantly lowered the phospho-AMPK/AMPK and phosphor-ACC/ACC ratio in the liver tissue. ACC phosphorylation is regulated by AMPK activity. Therefore, it appears that the high level of AMPK phosphorylation resulted in phosphorylation of ACC. As shown in Figure 10, the reduction of phospho-AMPK/AMPK and phospho-ACC/ACC ratio induced by high-fat diet was almost completely recovered by LE treatment. 

## 4. Discussion 

Obesity has become a major worldwide health problem [3], not least because it is associated with many diseases, particularly diabetes, hypertension, osteoarthritis, and heart disease. As a result, obesity has been found to reduce life expectancy, and causes huge economic and social problems [4–8]. Thus, the quest for possible natural products that aid in weight loss has been intensified. Intrigued by recent reports indicating that *Laminaria japonica* Areshoung possesses various biological functions including anti-inflammatory [19], antitumor [20], antitheroscloresis [21], and antidiabetic activity [22], we sought to investigate whether *Laminaria japonica* Areshoung has antiobesity effect. 

In this study, antiobesity activity of LE was investigated by measuring body weight gain, food intake, and lipid profiles in LE-treated obese rats. High-energy diets are widely used in nutritional experiments as a strategy to induce overweight conditions and fat deposition in animals [32]. We also observed that final body weight, body-weight gain, and total fat-pad weight in the HD group were greater by 30.1%, 65.9%, and 135.5%, respectively, compared to the ND group. Administration of LE (400 mg/kg) or tea catechin for 6 weeks remarkably decreased the body weight gain by more than 21% compared with that of the HD group (Table 3). The LE significantly and dose-dependently decreased food intake and food efficiency ratio. The epididymal, abdominal, visceral, and brown fat-pad weights of the obese rats receiving LE (200 or 400 mg/kg) proved to be significantly lower compared to those of the HD group (Table 3). In addition, the plasma insulin level was decreased by LE or tea catechin treatment (Figure 4).

In general, a high-fat diet significantly increases the TC and TG levels in serum and liver [33]. Our data also showed that rats in the HD group exhibited significantly higher TG, TC, LDL-C and FFA levels, and lower HDL-C and HDL-C/TC ratio. However, the administration of LE or tea catechin reduced these parameters to near normal levels in serum and liver (Figures 3 and 5). These results indicate that oral administration of LE suppresses the accumulation of body fat in a dose-dependent manner, resulting in improved lipid profiles in serum and liver, and decrease of insulin level. Notably, LE treatment did not show any renal or hepatic toxicity (Table 4). 

Blood levels of leptin, which is a key fat-derived regulator of appetite and energy expenditure, normally correlate positively with the extent of the TG stores in adipocytes [34, 35]. In our study, the plasma leptin level was decreased by LE or tea catechin treatment (Figure 4). Moreover, our histological examinations revealed that the sizes of the adipocytes were significantly reduced in LE-treated rats (Figure 2). These results suggest that the decreased plasma leptin levels after LE supplementation might be attributable to decreased lipid accumulation in white adipose tissue. In addition, HD is known to increase the synthesis of fatty acids in the liver and the delivery of free fatty acids to the liver [36, 37], and decrease *β*-oxidation of free fatty acids, resulting in fat accumulation in the liver [35]. Our histological examination also showed macrovesicular steatosis in liver tissues of the HD group (Figure 6). However, LE supplementation noticeably attenuated the extent of steatosis, suggesting that LE may regulate lipid storage and mobilization in adipocytes by modulation of the leptin level.

It is noticeable that LE (400 mg/kg) treatment significantly reduced the serum glucose, and insulin levels in obese rats (Figure 4). It is well known that *Laminaria japonica* reduces the intestinal absorption of glucose. Considering that the increase in blood glucose and insulin in animals on the high-fat diet is a strong indicator of obesity-induced insulin resistance and progression to type-2 diabetes, these results suggest that LE has protective effect against the development of obesity-induced insulin resistance.

It has been reported that TNF-*α* secretions are elevated by the accumulation of fat in adipocytes and that TNF-*α* induces insulin resistance in obese animal models [38–42]. The TNF-*α* level was greater in the HD group than in the ND group. LE (400 mg/kg) treatment reduced the serum TNF-*α* level to values similar to those of the ND group. Tea catechin also showed similar effects to LE at 100 mg/kg (Figure 4). It seems possible that the lowered TNF-*α* levels are due to the decreased fat mass by LE treatment. 

To gain insight into the molecular mechanism underlying the antiobesity effects of LE described above, we determined the expression of genes involved in obesity and lipid metabolism. PPAR*α* is known to regulate lipid metabolism through a ligand-dependent transcriptional activation of the expression of genes involved in the fatty acid oxidation pathway. Activation of PPAR*α* expression is known to increase expression of CPT-1, ACO, UCP2, and UCP3 to elevate energy expenditure, subsequently resulting in antiobesity actions [43–46]. The expression levels of PPAR*α* in the skeletal muscle showed significant increase in the rats fed with LE or tea catechin, compared to the HD group (Figure 7). The expression of downstream target genes of PPAR*α*, such as ACO, CPT1, UCP2 and UCP3 were significantly increased by LE administration. Furthermore, LE lowered the mRNA expression of ACC2, which is a potent inhibitor of fatty acid oxidation in the skeletal muscle. Decrease in ACC2 leads to a decrease in the cellular malonyl-CoA content, thereby activating carnitine palmitoyl transferase-1 (CPT-1) and increasing the transport of fatty acids into the mitochondria for *β*-oxidation. In this study, LE may lead to loss of body fat by increasing fatty oxidation in the skeletal muscle through inhibition of ACC2 (Figure 7). These results suggest that the antiobesity effect of LE is likely due to the increase of fatty oxidation and thermogenesis in the liver and skeletal muscle through PPAR*α* and its downstream target genes. 

To maintain lipid homeostasis, adipocytes perform two reciprocal biochemical processes, called lipogenesis and lipolysis. These two processes are regulated by hormones, lipid metabolites and nutritional conditions [47]. Regarding these signals, lipogenic transcription factors, including SREBP1c, play key roles in lipid metabolism in adipose tissue by regulating the gene expressions of enzymes for fatty acid synthesis and uptake and TG synthesis [47, 48]. FAS, ACC1, and SCD1, a downstream factor of SREBP1c, are enzymes for de novo fatty acid synthesis and energy homeostasis [49]. Fatty acids are critical substrates for biosynthesis of triglycerides. Several genes, especially GPAT, AGPAT, and DGAT, are involved in catalyzing the several steps of triglyceride synthesis in turn [50–55]. We demonstrated that the mRNA levels of lipogenesis-related genes such as SREBP1c, FAS, ACC1, and SCD1, which were increased in liver and epididymal white adipose tissue of rats fed with HD, were downregulated when they were fed with LE (200 mg/kg) or tea catechin (Figure 8). Similarly, the mRNA levels of GPAT, DGAT1, and AGPAT were decreased in the liver from rats treated with LE or tea catechin, compared with the HD group. 

Furthermore, LE lowered the mRNA expression of PPAR*γ* and LPL, which are related to fat intake by epididymal white adipose tissue (Figure 9). Reduced expressions of fatty acid synthetase [9], PPAR*γ* [56, 57], and LPL [58] in adipose tissue suppress the onset of obesity. Conversely, LE increased the mRNA expression of ATGL, HSL, and CPT1, which are related to lipolysis in epididymal white adipose tissue. These results suggest that the antiobesity effects of LE are possibly due to the suppression of the mRNA expression levels of genes involved in fatty acid and TG synthesis.

To further investigate the molecular mechanism, we determined the effect of LE on the activity of AMPK. Both leptin and adiponectin stimulate fatty acid oxidation via the phosphorylation of AMPK and ACC [59, 60]. AMPK is a phylogenetically conserved serine/threonine protein kinase which has been proposed to act as a “metabolic master switch” mediating the cellular adaptation to environmental or nutritional stress factors [61]. Once activated, AMPK leads to a concomitant inhibition of energy-consuming biosynthetic pathways, such as fatty acid and sterol synthesis, and activation of ATP-producing catabolic pathways, such as fatty acid oxidation. Phosphorylated AMPK phosphorylates (inactivates) ACC and lowers the intracellular malonyl-CoA level, which is the substrate for fatty acid synthesis and, at the same time, the inhibitor of CPT-1, the rate-limiting enzyme of mitochondrial fatty acid oxidation [62]. Therefore, phosphorylated AMPK increases fatty acid oxidation through a decrease in myocardial malonyl-CoA levels and an increase in CPT-1 activity [63–65]. In the present study, the protein levels of phospho-AMPK and phospho-ACC, which were reduced in the liver of rats fed with HD, were upregulated when they were fed with LE (100 mg/kg) or tea catechin. Tea catechin also showed parallel effects to LE at 200 mg/kg (Figure 10). Thus, it appears that LE stimulates *β*-oxidation of fatty acid by activation of AMPK pathway.

## 5. Conclusion

We conclusively demonstrated that LE has beneficial effects for the suppression of high-fat-diet induced obesity. It provided evidence that LE supplementation decreases body weight gain, food intake, lipid levels in serum and liver, and size and number of adipocytes in HD-induced obese rats. LE appears to show such activities by modulating the lipid metabolism through the decreased activity in lipogenesis as well as the increase in fatty acid oxidation, suggesting that LE has excellent potential as an effective antiobesity agent with no obvious toxicity.

## Figures and Tables

**Table 1:** Composition of normal diet and high-fat diet.

Ingredient	Group	
	ND	HD
Casein	80.0	80.0
Cornstarch	60.0	60.0
Sucrose	200.0	122.6
Corn oil	45.0	0
Lard	0	219.2
AIN-76 mineral mix	0	0.0
AIN-76 vitamin mix	4.0	4.0
DL-methionine	1.2	1.2
Choline bitartrate	0	0
Energy		
kcal/100 g of diet	390.2	487.0
Calories from fat (%)	11.5	45.0

HD: high-fat diet; ND: normal diet.

**Table 2:** Primer sequences.

Target	Primer	Sequence (5′–3′)	Amplicon size (bp)
PPAR*α*	F	TGACCAGCCTGATGGAGTTA	239
	R	ACTGATAGTGGGATTCTTATTGGG	
PPAR*γ*	F	TTCAGTTTGGAGACTTCGGACC	183
	R	TAGGCTCCTGCCAGATTACTCC	
ATGL	F	CTCCCAAACTGACCCTTAAACG	194
	R	AGACACTTTCTCCCAGTGGACC	
HSL	F	GCTCCCATCGTCAAGAATC	262
	R	TAAAGCGAATGCGGTCC	
UCP1	F	CAGAGAGTTTGTCCTCTGGTGC	265
	R	GAAAAATCAAGGGTCCTGCCCC	
LPL	F	ATTCTGCACTGCGCAAAGTAC	181
	R	AGTAAAACAAAAGAGCGGCAA	
SCD-1	F	CACATCAACTTCACCACGTTCTTC	74
	R	GAAACTTTCTTCCGGTCGTAAGC	
CPT1 (muscle)	F	CAATGGTGGGATGAGAGAAC	175
	R	AACCCAGAACTTTAACCTCAA	
CPT1 (liver)	F	AACCTTGGCTGCGGTAAGACTA	184
	R	AGTGGGACATTCCTCTCTCAGG	
ACO	F	CCAACAAGGTGACATGCTGTGT	184
	R	ATTCGCATTGTGAGAGCAGTTC	
UCP3	F	GTTTTGCTGATCTCCTCACCTTT	242
	R	GAACTGCTTGACAGAGTCATAGAGG	
SREBP-1c	F	GATGCCAACCAGATTCCCTAAG	210
	R	TCAGTTGTTTCTTTGCCTTCCA	
ACC-1	F	TCTATTCGGGGTGACTTTC	109
	R	CTATCAGTCTGTCCAGCCC	
ACC-2	F	GGAACTCACGCAGTTGACCAGG	299
	R	CACATAAACCTCCAGGGACGCC	
FAS	F	CTGGACTCGCTCATGGGTG	111
	R	CATTTCCTGAAGTTTCCGCAG	
DGAT	F	AGACTAGGAGGAGTGTGCAGGC	212
	R	CGCTTCTTCCAAGGGAACTATG	
AGPAT	F	GCATTTCAGGATCTCGTTCACA	197
	R	ATCAACCCAACGAGAGCACTTT	
GPAT	F	TGATCAGCCAGGAGCAGCTG	508
	R	AGACAGTATGTGGCACTCTC	
UCP2	F	GCATTGCAGATCTCATCACTTTCC	222
	R	AGCCCTTGACTCTCCCCTTG	
*β*-actin	F	GCTCTTTTCCAGCCTTCCTT	259
	R	TGATCCACATCTGCTGGAAG	

PPAR*α*: peroxisome proliferator-activated receptor *α*; PPAR*γ*: peroxisome proliferator-activated receptor *γ*; ATGL: adipose triglyceride lipase; HSL: hormone sensitive lipase; UCP1: uncoupling protein 1; SCD-1: stearoyl-CoA desaturase-1; CPT-1: carnitine palmitoyltransferase-1; ACO: acyl CoA oxidase; UCP3: uncoupling protein 3; SREBP-1c: sterol regulatory element-binding protein-1c; ACC1: acetyl-CoA carboxylase1; ACC2: acetyl-CoA carboxylase2; FAS: fatty acid synthase; DGAT: diacylglycerol O-acyltransferase; AGPAT: acylglycerol-3-phosphate-O-acyltransferase; GPAT: glycerol-3-phosphate-acyltransferase; UCP2: uncoupling protein 2.

**Table 3:** Effects of the LE on body weight, food efficiency ratio, and fat-pad weights in HD-induced obese rats.

Group	ND	HD	HD + tea	HD + LE100	HD + LE200	HD + LE400
Initial body weight (g)	453.33 ± 30.95	553.25 ± 35.12^###^	553.00 ± 41.79	553.20 ± 38.40	553.20 ± 41.32	553.20 ± 33.80
Final body weight (g)	560.40 ± 16.94	729.13 ± 77.27^###^	631.13 ± 42.13**	692.20 ± 24.53	665.60 ± 34.88*	634.00 ± 16.18*
Body weight gain (g)	106.00 ± 19.89	175.88 ± 83.91^#^	93.29 ± 41.32**	139.00 ± 42.34	112.40 ± 27.26**	93.50 ± 24.38***
Food intake (g/rat/day)	27.02 ± 1.88	19.88 ± 1.24^###^	16.60 ± 1.66*	17.88 ± 1.78	17.57 ± 1.56	17.06 ± 0.99*
FER (%)	9.55 ± 1.54	17.48 ± 3.82^#^	11.50 ± 1.83*	15.02 ± 2.99	14.76 ± 3.97	10.71 ± 2.85*
Whole fat (g)	32.61 ± 1.77	76.79 ± 5.17	59.73 ± 4.17	71.94 ± 3.26	66.38 ± 2.99	60.13 ± 2.77
Abdominal (g)	14.73 ± 3.44	39.35 ± 8.37^###^	29.88 ± 4.74	36.48 ± 2.64	33.59 ± 3.94	31.45 ± 4.38
Epididymal (g)	10.94 ± 2.39	23.39 ± 7.15^#^	19.72 ± 9.13	22.06 ± 6.06	21.74 ± 4.27	19.15 ± 4.08
Visceral (g)	6.50 ± 1.18	13.39 ± 5.09^#^	9.54 ± 2.80	12.86 ± 4.24	10.51 ± 3.71	8.99 ± 2.56
Brown (g)	0.44 ± 0.08	0.67 ± 0.09^###^	0.59 ± 0.02	0.55 ± 0.08	0.54 ± 0.05	0.54 ± 0.06

Data are mean ± SD values (*n* ≥ 8). ^#^ *P* < .05, ^###^ *P* < .001 versus the ND group; *P < .05, **P < .01, ***P < .001 versus HD groups. FER: food efficiency ratio.

**Table 4:** Evaluation of the LE on the liver and renal functions in HD-induced obese rats.

Group	ND	HD	HD + tea	HD + LE100	HD + LE200	HD + LE400
GPT (IU/L)	53.72 ± 0.52	56.35 ± 1.84^#^	54.83 ± 1.30	57.35 ± 2.10	54.38 ± 6.68	54.96 ± 5.16
GOT (IU/L)	76.00 ± 0.58	77.60 ± 0.48^##^	75.44 ± 0.56***	76.69 ± 1.07	76.58 ± 0.88*	76.41 ± 0.70*
BUN (mg/dL)	16.43 ± 0.23	19.71 ± 0.93^###^	15.98 ± 0.73***	16.7 ± 1.21***	15.59 ± 1.34***	15.42 ± 0.36***

Data are mean ± SD values (*n* ≥ 8). ^#^ *P* < .05, ^##^ *P* < .01, ^###^ *P* < .001 versus the ND group; *P < .05, ***P < .001 versus HD groups. GPT: glutamate pyruvate transaminase; GOT: glutamic oxaloacetic transaminase; BUN: blood urea.
